# “I care about sex, I care about my health”: A mixed-methods pre-test of a HIV prevention mobile health app for Black women in the southern United States

**DOI:** 10.1371/journal.pone.0289884

**Published:** 2023-10-18

**Authors:** Rasheeta Chandler, Dominique Guillaume, Sherilyn Francis, Eric Xue, Kewal Shah, Andrea Parker, Natalie Hernandez

**Affiliations:** 1 Nell Hodgson Woodruff School of Nursing, Emory University, Atlanta, GA, United States of America; 2 Center for Infectious Disease and Nursing Innovation, School of Nursing, Johns Hopkins University, Baltimore, MD, United States of America; 3 School of Interactive Computing, College of Computing, Georgia Institute of Technology, Atlanta, Georgia, United States of America; 4 Center for Maternal Health Equity, Community Health and Preventive Medicine, Morehouse School of Medicine, Atlanta, Georgia, United States of America; Florida Atlantic University Charles E Schmidt College of Medicine, UNITED STATES

## Abstract

**Background:**

Black women experience higher rates of adverse sexual and reproductive health and HIV outcomes, however the use of mHealth to address these health disparities in this population has been inadequate. This study involved a one-month pre-test with Black women living in metro-Atlanta to evaluate the usability, acceptability, and engagement of an HIV prevention app *SavvyHER*.

**Methods:**

An explanatory mixed-methods design was employed in which quantitative data was collected through weekly cross-sectional surveys, and qualitative data was collected through semi-structured in-depth interviews. Descriptive and ANOVA analysis was conducted for the quantitative data using STATA software. Qualitative data was analyzed through qualitative descriptive methods on Atlas.ti.

**Results:**

Participants had high levels of acceptability towards the app and used *SavvyHER* moderately. The most frequently used features were live groups (2.96 ±0.22, 95% CI 2.51,3.41), viewing resources and educational information (2.77 ± 0.21, 95% CI 2.33,3.20), and mental health monitoring (2.73 ±0.21, 95% CI 2.29,3.12). The least used features were pregnancy symptom monitoring (1.92 ±0.27, 95% CI 1.38,2.47) and STI symptom monitoring (2.0 ±0.25, 95% CI 1.48,2.52). In qualitative interviews, several women discussed how the ability to engage in active discussions and join live sessions with other end-users was a favorable aspect of *SavvyHER*. Although the app’s primary focus was on sexual and reproductive health and HIV prevention, women were more likely to access mental health monitoring and physical activity monitoring features. Women expressed their fondness of the app design and interface as it was reflective of the diversity of Black women.

**Conclusion:**

Further research is needed to explore the efficacy in using SavvyHER and additional mHealth interventions to enhance Black women’s sexual and reproductive health and overall wellness.

## Introduction

Over the last decade, there has been a rapid expansion in mobile health (mHealth) programs to address health disparities, particularly in the context of HIV prevention. However, limited evidence exists on the efficacy of mHealth applications (apps) in improving HIV outcomes for Black cisgender women. This is concerning given that this group experiences higher rates of HIV and sexually transmitted infections (STIs) compared to women of other racial groups [1]. Studies have continuously reported that Black cisgender women experience lower HIV and sexual and reproductive health (SRH) knowledge, while simultaneously experiencing inequitable barriers in accessing health care [2–4]. For instance, in the context of HIV prevention, although PrEP (Pre-exposure prophylaxis) is 99% effective in preventing HIV when taken as prescribed, Black cisgender women are less likely to use PrEP despite their high HIV risk due to limited knowledge and lack of access [5–11].

Mobile health interventions may have considerable potential in improving HIV and SRH disparities for Black cisgender women. However, it is critical for these technologies to be developed in a manner that reflects Black women’s health needs and concerns, while also acknowledging social and cultural aspects that may be influential in guiding their health behaviors. Among studies that have used mHealth for various populations, numerous challenges have been reported in program design and delivery, resulting in the downstream effects of low levels of retaining users and difficulties with sustaining engagement. The lack of mHealth interventions for Black women that aim to reduce SRH and HIV disparities, coupled with inadequate inclusion of Black women in mHealth research overall has resulted in substantial gaps in understanding how to best design and implement mHealth programs for this priority community [12].

Our study consisted of a one-month pre-test using an explanatory mixed-methods design to evaluate usability, acceptability, and engagement of the *Savvy HER* (Sexual/HIV Health Electronic Empowerment Resource) app. *Savvy HER* was designed based off extensive formative work to promote SRH outcomes of Black cisgender women living in the southern United States, with a key focus on reducing HIV risk through increasing PrEP knowledge, awareness, and uptake [9, 13–15]. The data collected from this pre-test will be used to refine the *SavvyHER* app in a pilot feasibility trial, a prelude for a future larger-scale randomized control trial.

## Methods

### Ethics

This study was approved by Emory IRB (STUDY00002857). Written consent was obtained from study participants during the quantitative phase of the study in which surveys were administered. Verbal consent was obtained from study participants prior to conducting qualitative interviews. Both written and verbal consent was obtained from a member of the research team after participants were deemed eligible for the study.

### Overview of *SavvyHER* features

*SavvyHER* contains a variety of features that were developed per extensive formative work conducted with Black women [2, 9, 13]. Although the primary aim of the app is to reduce HIV burdens for Black women, the formative work conducted by the research team revealed that Black women preferred an app that contained health information beyond HIV prevention. Thus, the research team ensured that *SavvyHER* contained comprehensive health information and features that were accurate, timely, along with being culturally and contextually relevant. *SavvyHER* includes interactive stand-alone features, which are categorized into larger domains (My Logger, My Test, My Resources. My Circle) as described below.

My Logger enables participants to log and track health data. The health data that can be tracked includes mental health, menstrual cycle, physical activity, STI symptom monitoring, and pregnancy symptom monitoring data. Participants are also encouraged to journal about any sexual/reproductive health encounters and mental health concerns, appointments, indicators of social instability associated with HIV risk behaviors and descriptive anecdotes in the journal section of My Logger. My Logger integrates self-tracking data to elicit preventive health messaging, and incorporates symbolic modeling, behavioral knowledge, behavioral acceptance, and cultivation of supportive social networks.

My Test contains two components- an HIV resource locator and commodity ordering. Using the HIV.gov application programming interface (API) gateway, a GPS locator for HIV testing and PrEP clinics was incorporated into *SavvyHER* where participants can locate HIV-related services within their geographic area. The commodity ordering feature provides participants with the ability to order condoms, STI testing kits, and sexual pleasure items in which they were incentivized with through an in-app widget.

My Resources gives participants access to a multimedia resources consisting of news and content discussing health and wellness including topics pertaining to women’s health, mental health, reproductive health, HIV prevention, and other relevant health subjects using video clips, current events, infographics, news feeds, podcasts, and support groups.

My Circle consists of a virtual environment for end-users to connect, extending opportunities for peer modeling, learning, and support. Women have the opportunity to share sexual and reproductive health goals, and utilize messaging features to provide support around these goals. In addition, women have the ability to share insights they have found meaningful through the educational messaging components of the app to support peer learning. Throughout the pilot, moderated virtual groups discussing various SRH topics were hosted by the research team which consists of primary care and infectious disease clinicians, and public health specialists.

### Recruitment

Eligibility criteria consisted of the following: 18–45 years of age, self-identify as Black/African American, assigned female sex at birth and currently identify as a cisgender woman, PrEP qualified based on CDC criteria (i.e. sexually active with a partner of unknown or positive HIV status in the last six months), residence in high-risk areas for HIV in Georgia (i.e. Fulton, Cobb, Gwinnett, Dekalb counties), HIV negative status, and owning a smartphone.

Convenience sampling was used to recruit participants. Participants were recruited online through posting flyers on social media platforms (i.e. Facebook, Twitter, Instagram) and via the research team’s community partners. Upon meeting eligibility criteria, participants were consented and underwent a virtual enrollment session where they were provided with instructions by the study staff on how to download and use the app. Participants were encouraged to use all the features of the app during the 4-week study period.

### Measures

Demographic information (e.g. age, residence, relationship status, level of education) was provided by all participants upon study enrollment. Surveys adapted items from the system usability scale (SUS) which contained items measuring app use (i.e. How often do you use each of the following *SavvyHER* features), feature feedback (i.e. Thinking about your own sexual and reproductive health experiences, how helpful was each *SavyHER* feature in supporting your sexual and reproductive health needs?), app experience (i.e. Did you experience any problems when using app features?), and tech acceptance (i.e. Using *SavvyHER* improves my sexual and reproductive health experiences) [16]. Feasibility metrics were determined by meeting pre-defined benchmarks specific to retention, screening, and interaction with app features (e.g. weekly app sign-ins, time spent using the app as well as its features). App use and feature feedback were measured using a 5-point Likert scale (1-not at all used, 5-extremely used; 1-not at all helpful, 5-extremely helpful). App experience was measured using a nominal scale (yes, no, I did not use this feature).Tech acceptance was measured using a 7-point Likert scale (1-strongly disagree, 7-strongly agree). Semi-structured exit interviews explored participants’ overall experiences in using *SavvyHER*.

### Data collection

The pre-test consisted of four consecutive weeks of app use by a cohort of n = 10 Black women. Each week, reminders were sent to participants via phone calls and text messages to encourage women to engage with the app. Weekly surveys were sent to participants via text to assess usability and feature feedback. The overall acceptability of the app was measured during the final week of the pre-test. In addition, Google Analytics data was collected to evaluate app use in real-time, however this data is not presented in this manuscript. Participants were required to complete the one-month SavvyHER pre-test in order to participate in the qualitative phase. Exit interviews were conducted after week 4 to obtain descriptive details about users experience and their recommendations to improve the app. The qualitative data collected was used in the subsequent interpretation and clarification of results from the quantitative data analysis [17]. Undertaking these approaches enabled the research team to develop detailed and contextualized insights of app usability, acceptability, and feature feedback.

### Data analysis

Survey data were transferred from RedCap to STATA and Tableau for data analysis and visualization. Descriptive statistics (e.g. means, standard errors) were generated for all study variables. Weekly averages for app use, feature feedback, and tech acceptance were calculated, and used to generate a total weekly measure for each category. ANOVA analysis was conducted to compare averages in app use, feature feedback, and tech acceptance between each week. Statistical significance was set a p<0.05, however these values are solely provided for reference as our study was not adequately powered to detect significance.

Qualitative interview data was analyzed using Atlas.ti™ software. A qualitative descriptive approach was used, and transcripts were coded using open-coding methods followed by axial coding in which patterns amongst codes were identified, and codes were subsequently categorized into broader themes. Repetitive themes were used to reflect participant perspectives of the overall *SavvyHER* intervention coupled with perspectives regarding the specific components of the *SavvyHER* app [18].

## Results

A total of N = 10 participants enrolled in the study. Among the participants, n = 8 completed the quantitative and qualitative phases of the study. All participants identified as Black/African-American. The mean age of participants was 26.75, with ages ranging from 20–44. The majority of participants were single (n = 7, 87.5%) and had more than a high school level of education (n = 6, 75%) (Table 1).

**Table 1:** Participant characteristics.

Race	Ethnicity	Age	Residence	Marital Status	Education Level
African-American	Non-Hispanic or Latino	28	Cobb County	Single, but in a relationship	Post secondary education
African-American	Non-Hispanic or Latino	24	Dekalb County	Single, but in a relationship	Post secondary education
African-American	Non-Hispanic or Latino	20	Dekalb County	Single, never married	High school degree or GED
African-American	Non-Hispanic or Latino	24	Henry County	Single, never married	Post secondary education
African-American	Non-Hispanic or Latino	30	Fulton County	Single, never married	Post secondary education
African-American	Non-Hispanic or Latino	24	Dekalb County	Single, never married	Post secondary education
African-American	Non-Hispanic or Latino	20	Dekalb County	Single, never married	High school degree or GED
African-American	Non-Hispanic or Latino	44	Fulton County	Married	Post secondary education

### Feature feedback

Women in the study rated the *SavvyHER* features as being somewhat to very helpful. Feature preferences changed over the four weeks. In the first week, participants ranked the features in My Resources (3.83 ± 0.48, 95% CI 2.51,5.06), My Circle (3.67± 0.42, 95% CI 2.58,4.75), and My Test (3.67 ± 0.42, 95% CI 2.58,4.75) as being the most helpful. In week two, participants endorsed the My Logger menstrual cycle monitoring (4.0 ± 0.58, 95% CI 2.16,5.84) and physical activity monitoring (3.75 ± 0.75, 95% CI 1.36,6.14) as being the most helpful features, followed by My Resources (3.75 ± 0.48, 95% CI 2.23,5.27). My Resources was the highest rated feature in week 3 and week 4 (4.0 ± 0.32, 95% CI 3.12,4.89). The ANOVA analysis demonstrated that participants had higher ratings for all features in week 4 compared to week 1, however this was not statistically significant (p >0.05) (Table 2). Further details regarding feature feedback are provided below.

**Table 2:** Weekly averages of total app use, feature feedback, and tech acceptance.

Week	Total App Use		Feature Feedback		Tech Acceptance	
	*M* ± se	*95% CI*	*M* ± se	*95% CI*	*M* ± se	*95% CI*
Week 1	2.24 ± 0.38	1.46,3.01	3.25 ± 0.44	2.33,4.17	---	---
Week 2	2.09 ± 0.31	1.45, 2.73	3.23 ± 0.50	2.18,4.28	---	---
Week 3	2.27 ± 0.31	1.62,2.92	3.33± 0.31	2.69,3.97	---	---
Week 4	3.01 ± 0.42	2.14,3.87	3.70 ± 0.45	2.76,4.64	5.30 ± 0.63*	3.30,7.30*
*P-value*	p = 0.32		p = 0.85		---	

*Tech acceptance was only measured at week 4

#### My Logger

Across all four weeks, the My Logger features that were cited as being the most useful were physical activity monitoring (3.67 ± 0.27, 95% CI 3.10–4.23),menstrual cycle monitoring (3.67 ± 0.25, 95% CI 3.14–4.19), and mental health monitoring (3.57 ± 0.19, 95% CI 3.18–3.97). Pregnancy symptom monitoring was ranked as the least useful feature (3.48 ± 0.28, 95% CI 2.89–4.06) during the study period.

Nearly all interviewees described their appreciation of the mental health feature, and voiced favorable aspects regarding how *SavvyHER* provided different options for mood tracking: *“I liked the icon*, *just like the visual of ‘how are you feeling*?*’ Because sometimes*, *you know*, *advice around mental health it to you know*, *write out your feelings*, *things of that nature*. *Sometimes you [just] want to have like this very long*, *lengthy journal entry*, *and sometimes*, *[you want a] simple*, *‘how are you feeling in the moment*?*’ and seeing your emotions reflected back to you*.*”*

Although participants agreed that the menstrual tracker was a helpful feature, most women stated that they already had a menstrual tracker app on their phone they were committed to. As a result, several participants were not as motivated to use the menstrual tracker feature on SavvyHER. One participant who did use the SavvyHER menstrual cycle feature voiced how she appreciated the ability to be able to describe her flow level, as her current menstrual cycle app did not enable her to input flow level data: “*It [her current period tracker app] doesn’t have like*, *flow [measurements]*, *like for me to enter a flow level each day or so*. *I think that was a nice feature within this SavvyHER app that I didn’t think about*, *and that isn’t’ in the other app that I have*.*”*

All women in the study detailed how the STI tracking features was a benefit to their SRH. Several women were not sexually active during the time of the study, and therefore they stated that the STI tracking was not a feature they were actively using. However, women indicated that if they were to be sexually active and/or have multiple partners, then that would prompt them to use the feature. This participant provided insight on their perceptions regarding the usefulness of the STI tracking feature: *“I think it’s [STI tracking] important to use…if I was just like*, *more sexually active I would use it more just because it’s kinda like tracking in real time*. *So when you like look back on*, *hmmm things have been kind of weird*, *but you can you can see the history in real time*. *So I think it’s useful tool*.*”* Another participant discussed how the STI tracking feature was beneficial for monitoring for STI symptoms: *“It [the STI tracker] helps them [end-users] understand*, *like*, *if they’re able to see the symptoms that they have*, *or whatever*, *I guess it kind of helps them*. *basically*, *just keep a lookout and knowing when to get something checked out*.*”*

While most participants found the various trackers in the app to be useful, one participant voiced how they had difficulty continuously engaging with the trackers: *“My problem is I tend to fall off the bandwagon when it comes to like*, *you know*, *emotional trackers I tend to fall off the bandwagon for those*, *but I do think that they’re extremely helpful*. *for other people*.*”* When participants were asked what could be done to improve app usability, it was mentioned that including prompts or notifications to remind end-users about logging their sexual and reproductive health experiences would be helpful, as many participants had difficulty remembering to log their experiences. *“Sometimes it was hard to remember to log or just*, *you know*, *I think like*, *maybe like if the app had like a notification…you know*, *obviously*, *nobody wants like*, *continuous notifications*. *But just like a prompt would be good*. *Because sometimes you just get through the day*, *and I just like to forget*, *so it’d be nice if that was like a prompt*.*”*

#### My Test

Among the My Test features, participants ranked STI testing locations (3.29 ±0.29, 95% CI 2.69,3.89) as being more helpful than commodity ordering (3.0 ±0.30, 95% CI 2.37,3.63) with this finding remaining consistent across all four weeks.

In qualitative interviews, participants favored the STI locator and at-home commodity ordering functions of the app. They viewed the My Test features as being helpful in meeting their SRH needs, as vocalized by this participant: *“Especially with the [STI] locator…where you’re able to show numerous locations where you can go where they have STD testing and things like that*. *You have so many different locations around the city of Atlanta*. *It’s awesome because you know*, *it’s like*, *if you’re not in that lifestyle you don’t know …you just don’t know… but you all show different places [for STI testing]*.*”*

Another participant discusses the benefits of having access to at-home STI testing kits through commodity ordering, which is something she says she never experienced in apps that she previously used: *“[I preferred] the [at-home] STI test… you’ll never really just hear people saying*, *‘at home STI tests’ like you will have to go to a clinic*, *or you got to go to a doctor’s office or you got to go to the emergency room like Urgent Care*. *You never hear [about at-home STI tests] in my community or*, *you know*, *my circle…I think that’s awesome…what you all do with the app is 100% awesome*.”

The ability to order pleasure commodities and the promotion of pleasurable aspects of sexual health was a favorable aspect of the app for many women. “*So say we log into the app every day*, *you get like a finger thing [sexual pleasure commodity]…that’s something different that you never really hear about getting for free*. *Also*, *like*, *you know*, *we’re gonna masturbate in time*, *you want to try something different*? *You want to know*,*’ okay how did it feel*?*’ so it’s like*, *I will try that…why not*?*”*

#### My Resources

**Fig 1:**
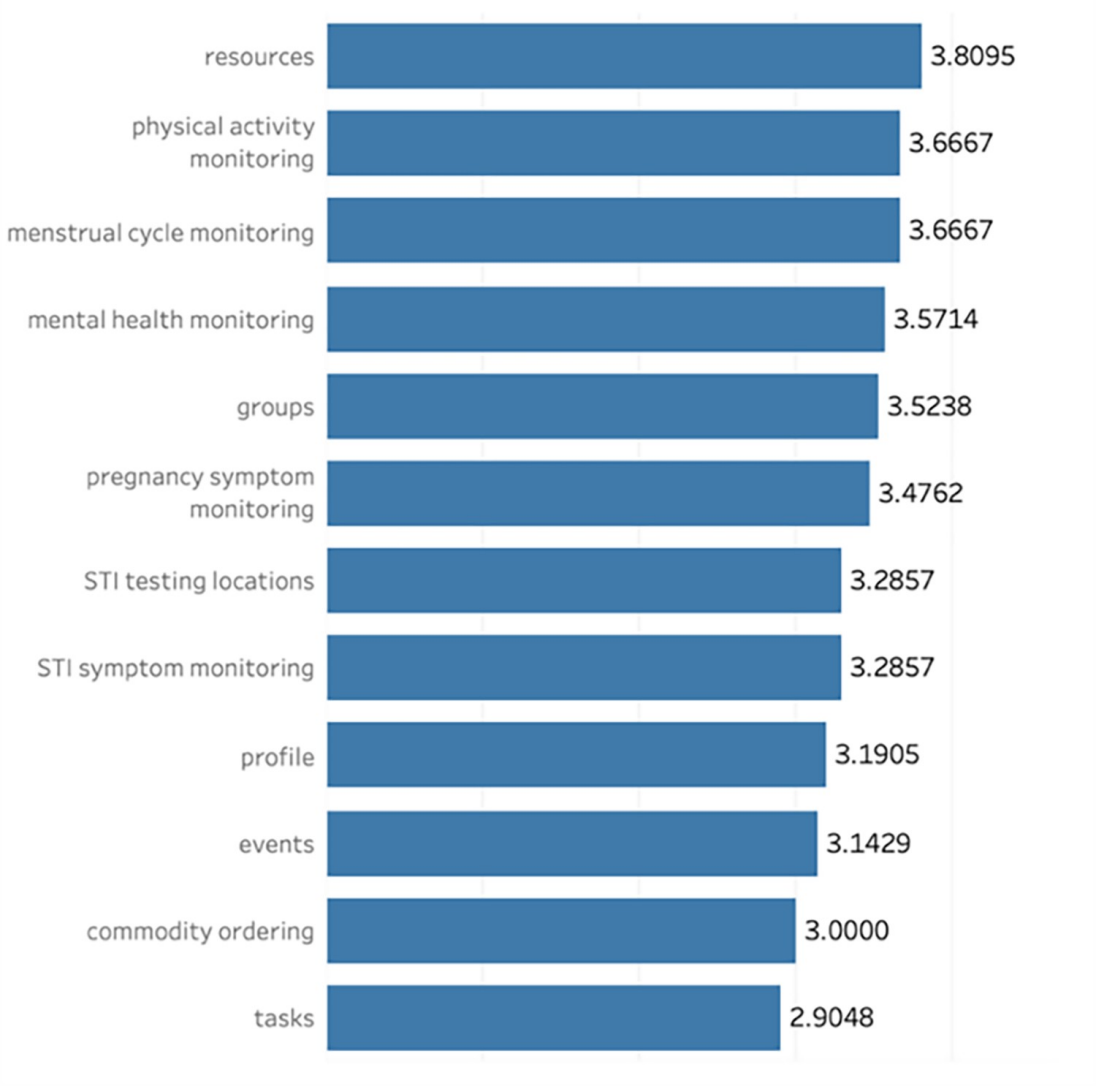
Feature helpfulness ratings averaged across four weeks.

Participants ranked My Resources as being the most helpful feature over the course of the pre-test (Fig 1). When comparing weekly differences, participants ranked the My Resources as being the most helpful in week 4 (4.0 ± 0.32, 95% CI 3.12,4.89) compared to all other weeks.

This participant mentioned that although at the time of the study they were not in need of resources, they appreciated knowing that they could access resources through the app and found the feature to be unique: *“I really looked at the map a couple of times*, *and I love the different pinpoints or the areas nearby that offered assistance if needed…but because at the time [of using the app] I didn’t feel like I needed any resources*, *I didn’t really dive deep into that*. *But I liked knowing that it was there*. *I thought that was really unique*. *That’s not something that I’ve seen before*. *And I think that’s great*, *especially for young black women*.*”*

Participants enjoyed that ability to react to posts on the Resources page and the ability to comment on posts as described by this participant: *“[I prefer] to allow people to comment on them [posts] because maybe something*, *maybe someone might write something that’s relatable*. *Or write something that shows their experience*, *something like that*, *and being able to engage with other people about it*, *and see what their thoughts were on it*. *Because I know I like to read comments on things that I view on social media… And even with articles on websites and stuff*, *I’m still looking at the comments*.*”*

The My Resources feature had components that were engaging in which participants could react to posts through emojis and comments. One participant described how they liked the engaging components of the feature, however they experienced technical issues which limited their use: *“I like the resources I know that sometimes*, *people you could respond to some of them [end-users] or [engage with] like the visual aids*, *but also sometimes*, *it was hard to click in like some other like if someone posted like a graphic*, *I couldn’t really open it*. *But I think like once I like would hit share or maybe save it to my phone and I could see it better*.*”*

#### My Circle

My Circle was ranked as being somewhat to very helpful (3.52 ± 0.18, 95% CI 3.15,3.89) over the study period. When comparing between weeks, users found My Circle to be the most helpful feature in week 4 (3.8 ±0.37, 95% CI 2.77,4.84).

In qualitative interviews, several women discussed how the ability to engage in active discussions with other end-users was a favorable aspect of *SavvyHER*. This participant described how My Circle enabled participants to discuss health topics that were relatable to the Black community: *“I can relate to [the group chats] and [the My Circle feature discusses] what I go through in my community… that’s something that I really like*, *and I really enjoy*. *And it’s about different stuff*. *It’s about everyday life stuff like sex-ed and sex health and things like that*. *I care about sex*, *I care about my health*. *So you all talk about that in the app*. *And that’s something that I can relate to*. *I really like that part of the app*.*”*

Participants provided rich description regarding their use of My Circle: *“I think the group feature was my favorite*. *And that was really the main thing I focused on*, *or interacted with the most just talking to other women*. *I think that’s what really makes the app felt feel like alive*. *And like there were other people who are looking for help or want to talk to people*. *So I really interacted with the groups especially*, *you know*, *the groups being catered towards specific topics like sex toys*, *or dating or STDs*. *I really liked that*, *that interaction and if I’m thinking about something specific*, *let me go find a group where am I find somebody who’s also talking about the same thing*.*”*

However, a common theme that arose during interviews were concerns pertaining to anonymity particularly when interacting with other end-users. One participant described how due to the lack of anonymity on the app, she was deterred from actively engaging in discussions pertaining to SRH: *“I think the group’s module was pretty good…some of the conversations that I did see*, *like*, *in the groups can be pretty*, *you know*, *might be a little…private*, *and obviously*, *it’s up to the user to engage in it*. *But because you can see other people’s names and stuff*, *I don’t know if I would like*, *obviously*, *it allows for that safe space…you can get to know people on a deeper level…but I don’t know if I would have necessarily [engaged with My circle] just because I could see names…”* Another participant stated that due to not knowing the other end-users, she was hesitant to engage with My Circle, and said that she is more comfortable discussing SRH topics with people she is familiar with as opposed to end-users whom she is unfamiliar with. *“I think for me*, *when I’m discussing*, *talking [about sexual and reproductive health] it’s like*, *easier to talk with people that you know*…*it’s not anonymity [that’s a barrier to engaging with My Circle]*, *but just the lack of familiarity with others…”*

One participant described how the content within virtual group education sessions helped to increase their sexual health awareness and knowledge: *“I feel like through the app*, *and through this whole course*, *I feel like I’ve learned a lot that I didn’t know…being able to buy sexual health products*, *or condoms off of it[the app]*, *or it having access to sexual awareness events*, *and to be able to see other women posting on the forum*, *and giving their advice or asking questions*. *I feel like it’s very helpful and insightful*.*”*

### App use and experience

**Fig 2:**
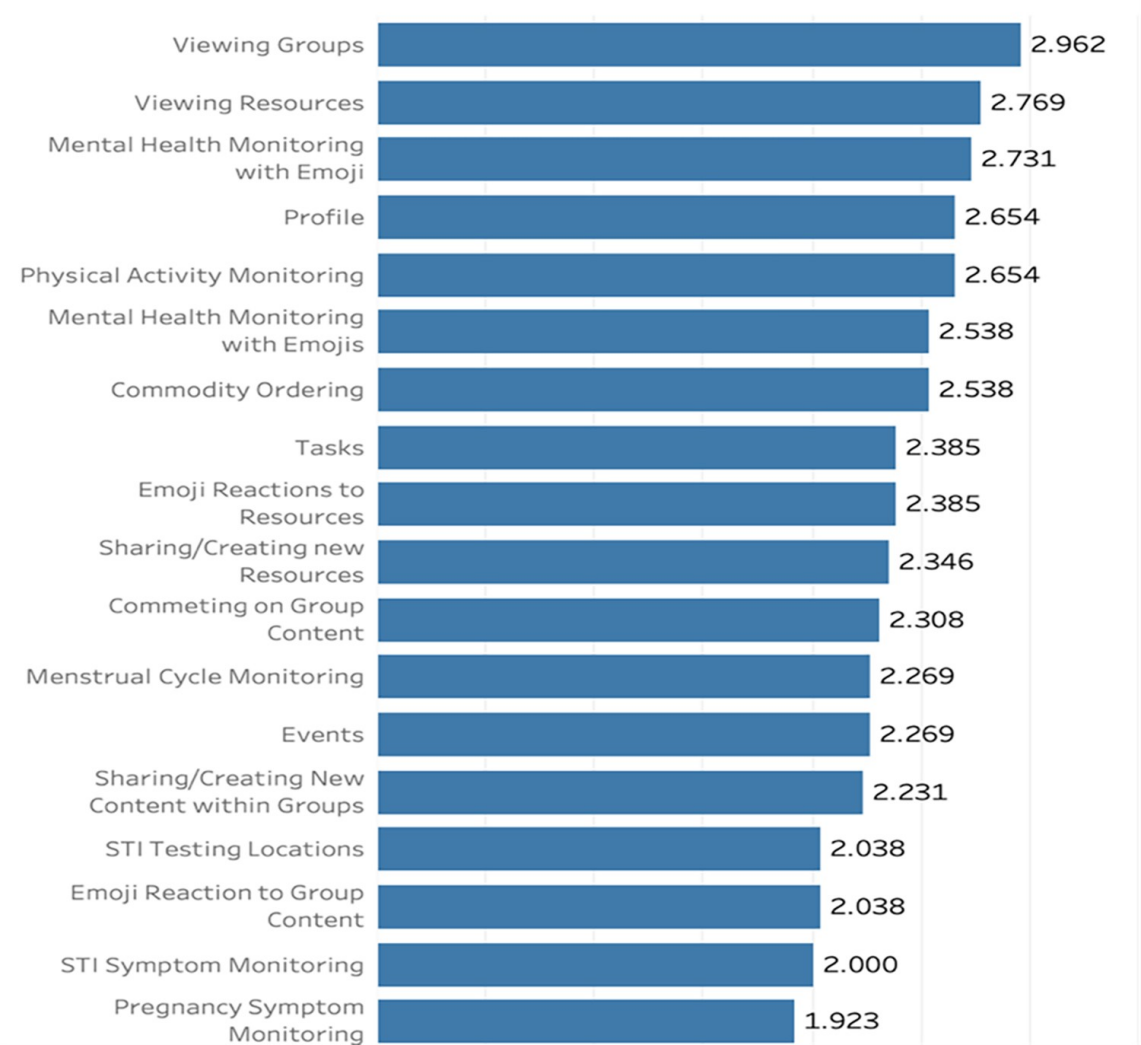
Feature usage averaged across four weeks.

Overall, participants were more likely to use app features that were not related to SRH and HIV. The most frequently used features were the live groups in My Circle (2.96 ±0.22, 95% CI 2.51,3.41), viewing resources and educational information in My Resources (2.77 ± 0.21, 95% CI 2.33,3.20), and mental health monitoring on My Logger (2.73 ±0.21, 95% CI 2.29,3.12). The features that were the least used included My Logger pregnancy symptom monitoring (1.92 ±0.27, 95% CI 1.38,2.47) and My Logger STI symptom monitoring (2.0 ±0.25, 95% CI 1.48,2.52) (Fig 2). There was a 34.38% increase in average total app use from week 1 (2.24 ±s 0.38, 95% CI 1.32,3.16) to week 4 (3.01 ± 0.42, 95% CI 1.92,4.09) however this finding was not statistically significant (p >0.05).

In qualitative interviews, participants voiced positive experiences in using *SavvyHER*. One participant described the app features that they used the most, which included the mental health tracker and the period tracker: *“I was staying more towards the mental health [feature] and I would have liked the period tracker*, *I think for the period tracker*, *that’s really important in terms of women’s sexual health*. *You know*, *just to keep track of when you’re having sex and all of that and…your symptoms whether or not it [sex] was safe or not… I think that’s important to be able to track that there*. *So I think that is a good functionality for the app*.*”*

Another participant explained how they enjoyed the live group sessions on My Circle, and how the app features supported engagement and positively contributed to her experience in using *SavvyHER*: “*I really liked the live session*, *that was one of my favorite things about the experience*, *to be honest…I really do like the substance and depth…when you’re having those engaging conversations*, *I love that sort of stuff…I was actively engaged*, *you know*, *sharing my*, *my experiences and responding*. *I like the live feature*.*”*

While the groups in My Circle was one of the most used features in the quantitative analysis, two participants in the qualitative interviews described how they did not engage with the groups function as they do not tend to seek live discussion and interaction within apps in general: *“I didn’t really engage with the groups [feature] that much*. *To be honest*, *I didn’t use that functionality at all*… *I don’t in general seek that feature in apps*. *I’m not even on social media*. *When I’m using technology*, *I’m more so using it for the content…not really using it for the engagement with other people*. *I’m more of a*, *like a forum reader…engaging with other users isn’t really at the forefront of my mind*.*”*

Regarding usability, several participants mentioned technical difficulties when trying to engage with app features, and also provided recommendations in improving the features:

*“Sometimes I would get the Resource tab not showing anything*. *And I think it’d be great to not constantly be logged out and having to sign back in every time*. *Because I asked to send a code to my phone*, *and then I don’t get it sometimes that happens*.*”* Another participant described recommendations on improving the app usability specifically for the mental health tracker: *“Whenever I enter a mental health blog*, *I realized I wasn’t able to view my previous ones*. *And I think it’d be a good idea to like*, *keep it history of them*. *So that we*, *whoever’s in use of it*, *could go back and review it or at least look at it*. *I think the mental health section should also have more mood options*. *I found that sound like sometimes I wasn’t able to find one that specifically fit*, *or something that makes sense*…*”*

### Tech acceptance

Tech acceptance was only measured during week 4. Total tech acceptance was high among participants (5.30 ±0.63, 95% CI 3.30,7.30). Participants agreed that *SavvyHER* was beneficial in improving their SRH experiences, mental health, physical health, along with social connectedness with other women. More specifically, women agreed that *SavvyHER* made their SRH experiences easier (5.25± 0.75, 95% CI 2.86,7.64) and enhanced their ability to get support from their social network (5.5 ± 0.65, 95% CI 3.45,7.56). Participants also endorsed that *SavvyHER* enabled them to monitor their sexual health (5.75 ± 0.63, 95% CI 3.75,7.75) better than apps they were currently using. Participants agreed that the app was easy to learn and use, and that the features were clear and understandable (5.0 ±0.58, 95% CI 3.16,6.84).

Participants compared *SavvyHER* to other available apps that focus on SRH topics. This participant specifically describes how in comparison to other apps, they appreciated that *SavvyHER* is designed for the needs of Black women: *“So I downloaded this app called Flo*..*Flo is a really cool app*, *but it’s not black women… with your app*, *it’s about black women*. *It’s about*, *what we go through*, *you [SavvyHER] are on like a more personal side that I can relate to*, *and what I go through in my community is something that I really like*, *and I really enjoy…and it’s*, *it’s about different stuff…it’s about everyday life stuff like sex-ed and sex health and things like that*.*”*

This participant described their experiences in using the *SavvyHER* app, and emphasized that they were encouraged to use it over the long-term: *“I really*, *really liked the app*. *This will be something that I will keep on my phone*. *Because it has so much in the app… I want to be able to want to track my period*. *I always always forget [to track my period]…I want to remember ‘Okay*, *when was the last time I had a cycle*?*’ I need to make sure that I’m up on it*. *I want to be able to remember okay*, *my cycle is about to start or my cycle didn’t start…I just want to be on top of it*.*”* The sense of community instilled on the app through the ability to engage with other end-users was a key motivator in using *SavvyHER for women*. This was found to be a unique aspect as described by one participant: *“But that’s what I really loved [referring to the interactive features of the app] I thought that was helpful in that that really provided a wonderful sense of community… it provides a sense of community…it’s a very live in real interaction*. *Even though it was structured*, *it still had organic elements to it*, *and I really loved that*.*”*

Women expressed their fondness of the app design and interface as it was reflective of Black women, whilst promoting diversity among Black women: *“I think it [the app design] doesn’t look so sterile and medical*. *It’s very*, *it’s very welcoming… I really liked the representation*, *like how*, *you know*, *there’s not a specific certain looking black woman*, *I’m seeing a lot of different faces*. *And I think that’s very nice to see representation*. *I think the interface is very well done*.*”*

## Discussion

This pilot study evaluated the feasibility, usability, and acceptability of *SavvyHER* among young adult Black women. Our findings demonstrate high levels of acceptability towards the app, with women endorsing *SavvyHER*’s features. These results corroborate findings from prior studies, in which Black women have been found to have high levels of acceptability of digital health interventions, especially when such interventions reflect their needs and lived experiences [14, 19–21]. While *SavvyHER* has a primary focus on HIV prevention, the researchers included content beyond HIV prevention based on the data obtained from their formative work with young adult Black women. Of note, our quantitative and qualitative results showcased that end-users had higher levels of engagement with app features that did not pertain to HIV prevention, particularly the mental health features. This finding is important to highlight, as this substantiates the need for the development of digital health interventions that are aligned with community needs and priorities. In many instances, digital health interventions do not accurately reflect the needs of communities and researchers develop tools that do not accurately resemble the communities they are trying to help [22]. The inclusion of information that promotes comprehensive health, wellness, and social connectivity, may encourage higher levels of engagement with the app as opposed to including HIV prevention information alone. This is critical to emphasize as numerous studies have indicated that young adult Black women harbor low levels of risk perception towards HIV [9, 11, 23–25]. Therefore, they may not be as compelled to download an app that solely includes content pertaining to HIV prevention. Incorporating additional health topics in addition to HIV prevention through digital health platforms may be an effective strategy in promoting HIV prevention and overall wellness for this community.

Participants in our study agreed that *SavvyHER* captured their attention through the design, interface, and content, and voiced that it would be an app they would use long-term. Often, HIV and STI mobile apps fail to attract the attention and positive reviews of target audiences, due to a combination of inadequate app promotion and failure to create engaging content; consequently resulting in low levels of uptake, limited downloads, and low user ratings [26]. App engagement often fluctuates, and smartphone owners regularly remove apps from their phones. This further emphasizes the need for the design, content, and features of apps to be both useful and entertaining for consumers to promote use [26]. Of note, many women in our study did not readily engage with the My Logger pregnancy monitoring and STI monitoring as those features were not relevant to their needs at the time of the study. More women however used the My Logger mood tracker as that appeared to be more pertinent to their health needs.

Individuals’ health and behaviors are not stagnant and vary across a continuum. This may reflect the need for researchers and developers of mHealth apps to include more comprehensive material as opposed to focusing on specific disease states in order to increase engagement over the long-term. A systematic review conducted by Muessig et al. (2013) reported that the majority of HIV-related apps lack psychological and emotional support resources and messages, along with active tools for self-monitoring [26]. *SavvyHER* included interactive components in which women were able to participate in virtual groups and interact with other end-users in real-time. In addition, personalized components were readily incorporated in which women had the ability to input data to track their SRH and mental health. Furthermore, end-users had the ability to order commodities such as at-home STI testing kits and pleasurable sexual health items which were highly favored by participants.

Public health interventions that focus on HIV prevention and SRH often neglect to promote positive aspects of sex and wellness. Sex positivity is largely overlooked for Black women, with sexual expressions often being stigmatized and marginalized [27, 28]. Thus, Black women are often surrounded by negative images and portrayals of how they should behave sexually, with such social influences having a detrimental effect on their overall SRH wellness and engagement in risk reduction behaviors [29]. Successfully addressing HIV and broader SRH disparities will require acknowledging these barriers through sex-positive and culturally-centered approaches. The features included within *SavvyHER* can support Black women’s sexual self-efficacy and heighten sexual agency, which is essential as studies have reported that one’s perception of sexual self-efficacy is a significant predictor of risk sexual behavior than knowledge of safe sex practices [29, 30]. To our knowledge, our app is the first developed for Black women that includes such interactive and personalized features.

### Limitations

Our study is not without limitations. The sample size was small thus it can be argued that these results cannot be generalizable to other communities, however the researchers were intentional in keeping a limited sample size s given that this was a pre-test and we were not aiming to establish statistical significance. Another limitation is that given the design of the study and recruitment methods, it is possible that selection bias was present. In addition, social desirability bias may have impacted the participant responses. However, this was mitigated through having data collectors who were properly trained in interviewing techniques. Lastly, while Google analytics data was collected to provide an objective measure of app usage in our study, our research team were unable to disaggregate the google analytics data between the study participants and the moderators who were simultaneously accessing *SavvyHER*. In our future RCT, we will ensure that data between participants and moderators is able to be disaggregated prior to study commencement.

### Future research

The research study elicited Black women’s perspectives regarding an app focused on providing HIV prevention content to Black women. Considerably more work will need to be done to further explore the efficacy of the *SavvyHER* app in improving Black women’s SRH and HIV outcomes. Using the data obtained from this pre-test, the research team will adjust the content and features of *SavvyHER*, and conduct a pilot feasibility randomized control trial. The randomized control trial will consist of comparing the *SavvyHER* intervention to a control arm that consists of a standard of care (e.g. HIV and STI counseling with a health care provider). The primary outcome measures for the RCT will be assessing feasibility, acceptability, and usability of the intervention compared to the control using a larger sample to establish statistical significance, along with comparing PrEP uptake amongst both arms. In the future iterations of *SavvyHER*, the research team anticipates enhancing the app’s features while also including novel components that promote patient-health care provider communication through creating clinician-specific functions focused on responding to health concerns for this priority community.

## Supporting information

S1 File Data dictionary codebook. (PDF)

S1 Data (XLSX)
