# Supplementary material for: “I care about sex, I care about my health”: A mixed-methods pre-test of a HIV prevention mobile health app for Black women in the southern United States
Source: PLoS One. 2023 Oct 18;18(10):e0289884. doi: 10.1371/journal.pone.0289884 (PMC10584133; doi:10.1371/journal.pone.0289884)
Supplement: S1 File — (PDF) [file pone.0289884.s001.pdf]

Data Dictionary Codebook

09/07/2022 2:21pm

	#	Variable / Field Name	Field Label<br>*Field Note*	Field Attributes (Field Type, Validation, Choices, Calculations, etc.)
Instrument: **Demographic** (demographic) Enabled as survey				
	1	[record_id]	Record ID	text
	2	[age]	How old are you?	text (number, Min: 18, Max: 50)
	3	[ycls2]	Are you Hispanic or Latino?	radio<br>1 Yes, Hispanic or Latino<br>2 No, not Hispanic or Latino<br>66 I choose not to answer this question
	4	[residence]	Where do you live?	radio<br>1 Fulton County, GA<br>2 Cobb County, GA<br>3 DeKalb County, GA<br>4 Gwinnett County, GA<br>88 Other, please specify {residence_other}
	5	[residence_other]<br>Show the field ONLY if:<br>[residence] = '88'	Please describe "other":	text
	6	[marital]	What is your relationship status?	radio<br>1 Married<br>2 Widowed<br>3 Divorced<br>4 Separated<br>5 In a domestic partnership or civil union<br>6 Single, but in a relationship with a significant other<br>7 Single, never married
	7	[ycls8]	What is the highest level of school that you have finished?	radio<br>1 Less than high school degree<br>2 High school degree or GED<br>3 More than high school degree<br>66 I choose not to answer this question
	8	[demographic_complete]	Section Header: *Form Status*<br>Complete?	dropdown<br>0 Incomplete<br>1 Unverified<br>2 Complete
Instrument: **App Use** (app_use) Enabled as survey				
	9	[app_use1]	Section Header: *APP USAGE How often do you use each of the following SavvyHER features:*<br>Profile	radio (Matrix)<br>1 Not at all used<br>2 Slightly used<br>3 Moderately used<br>4 Very much used<br>5 Extremely used

	10	[app_use2]	Viewing resources	radio (Matrix)<br>1 Not at all used<br>2 Slightly used<br>3 Moderately used<br>4 Very much used<br>5 Extremely used
	11	[app_use3]	Sharing/Creating new resources	radio (Matrix)<br>1 Not at all used<br>2 Slightly used<br>3 Moderately used<br>4 Very much used<br>5 Extremely used
	12	[app_use4]	Emoji reactions to resources	radio (Matrix)<br>1 Not at all used<br>2 Slightly used<br>3 Moderately used<br>4 Very much used<br>5 Extremely used
	13	[app_use5]	Viewing groups	radio (Matrix)<br>1 Not at all used<br>2 Slightly used<br>3 Moderately used<br>4 Very much used<br>5 Extremely used
	14	[app_use6]	Sharing/Creating new content within groups	radio (Matrix)<br>1 Not at all used<br>2 Slightly used<br>3 Moderately used<br>4 Very much used<br>5 Extremely used
	15	[app_use7]	Commenting on group content	radio (Matrix)<br>1 Not at all used<br>2 Slightly used<br>3 Moderately used<br>4 Very much used<br>5 Extremely used
	16	[app_use8]	Emoji reaction to group content	radio (Matrix)<br>1 Not at all used<br>2 Slightly used<br>3 Moderately used<br>4 Very much used<br>5 Extremely used
	17	[app_use9]	Mental health monitoring with emojis	radio (Matrix)<br>1 Not at all used<br>2 Slightly used<br>3 Moderately used<br>4 Very much used<br>5 Extremely used

	18	[app_use10]	Mental health journaling	radio (Matrix)<br>1 Not at all used<br>2 Slightly used<br>3 Moderately used<br>4 Very much used<br>5 Extremely used
	19	[app_use11]	Menstrual cycle monitoring	radio (Matrix)<br>1 Not at all used<br>2 Slightly used<br>3 Moderately used<br>4 Very much used<br>5 Extremely used
	20	[app_use12]	Physical activity monitoring	radio (Matrix)<br>1 Not at all used<br>2 Slightly used<br>3 Moderately used<br>4 Very much used<br>5 Extremely used
	21	[app_use13]	Tasks	radio (Matrix)<br>1 Not at all used<br>2 Slightly used<br>3 Moderately used<br>4 Very much used<br>5 Extremely used
	22	[app_use14]	Commodity ordering	radio (Matrix)<br>1 Not at all used<br>2 Slightly used<br>3 Moderately used<br>4 Very much used<br>5 Extremely used
	23	[app_use15]	Pregnancy symptom monitoring	radio (Matrix)<br>1 Not at all used<br>2 Slightly used<br>3 Moderately used<br>4 Very much used<br>5 Extremely used
	24	[app_use16]	STI symptom monitoring	radio (Matrix)<br>1 Not at all used<br>2 Slightly used<br>3 Moderately used<br>4 Very much used<br>5 Extremely used
	25	[app_use17]	STI testing locations	radio (Matrix)<br>1 Not at all used<br>2 Slightly used<br>3 Moderately used<br>4 Very much used<br>5 Extremely used

	26	[app_use18]	Events	radio (Matrix)<br>1 Not at all used<br>2 Slightly used<br>3 Moderately used<br>4 Very much used<br>5 Extremely used
	27	[app_use_complete]	Section Header: *Form Status*<br>Complete?	dropdown<br>0 Incomplete<br>1 Unverified<br>2 Complete
Instrument: **Feature Feedback** (feature_feedback) Enabled as survey				
	28	[f_fdbk1]	Section Header: *FEATURES FEEDBACK Thinking about your own sexual and reproductive health experiences, how HELPFUL was each SavvyHER feature in supporting your sexual and reproductive health needs?*<br>Profile	radio (Matrix)<br>1 Not at all helpful<br>2 Not so helpful<br>3 Somewhat helpful<br>4 Very helpful<br>5 Extremely helpful
	29	[f_fdbk2]	Resources	radio (Matrix)<br>1 Not at all helpful<br>2 Not so helpful<br>3 Somewhat helpful<br>4 Very helpful<br>5 Extremely helpful
	30	[f_fdbk3]	Groups	radio (Matrix)<br>1 Not at all helpful<br>2 Not so helpful<br>3 Somewhat helpful<br>4 Very helpful<br>5 Extremely helpful
	31	[f_fdbk4]	Mental health monitoring	radio (Matrix)<br>1 Not at all helpful<br>2 Not so helpful<br>3 Somewhat helpful<br>4 Very helpful<br>5 Extremely helpful
	32	[f_fdbk5]	Menstrual cycle monitoring	radio (Matrix)<br>1 Not at all helpful<br>2 Not so helpful<br>3 Somewhat helpful<br>4 Very helpful<br>5 Extremely helpful
	33	[f_fdbk6]	Physical activity monitoring	radio (Matrix)<br>1 Not at all helpful<br>2 Not so helpful<br>3 Somewhat helpful<br>4 Very helpful<br>5 Extremely helpful

	34	[f_fdbk7]	Pregnancy symptom monitoring	radio (Matrix)<br>1 Not at all helpful<br>2 Not so helpful<br>3 Somewhat helpful<br>4 Very helpful<br>5 Extremely helpful
	35	[f_fdbk8]	STI symptom monitoring	radio (Matrix)<br>1 Not at all helpful<br>2 Not so helpful<br>3 Somewhat helpful<br>4 Very helpful<br>5 Extremely helpful
	36	[f_fdbk9]	Tasks	radio (Matrix)<br>1 Not at all helpful<br>2 Not so helpful<br>3 Somewhat helpful<br>4 Very helpful<br>5 Extremely helpful
	37	[f_fdbk10]	Commodity ordering	radio (Matrix)<br>1 Not at all helpful<br>2 Not so helpful<br>3 Somewhat helpful<br>4 Very helpful<br>5 Extremely helpful
	38	[f_fdbk11]	STI testing locations	radio (Matrix)<br>1 Not at all helpful<br>2 Not so helpful<br>3 Somewhat helpful<br>4 Very helpful<br>5 Extremely helpful
	39	[f_fdbk12]	Events	radio (Matrix)<br>1 Not at all helpful<br>2 Not so helpful<br>3 Somewhat helpful<br>4 Very helpful<br>5 Extremely helpful
	40	[feature_feedback_complete]	Section Header: *Form Status*<br>Complete?	dropdown<br>0 Incomplete<br>1 Unverified<br>2 Complete
Instrument: **App Experience** (app_experience) Enabled as survey				
	41	[exp1]	Section Header: *Think about your experiences using SavvyHER. If there were any problems you faced when using each part of the app, please describe them.*<br>A) Did you experience any problems when using the MENTAL HEALTH section of the app?	radio (Matrix)<br>1 Yes<br>0 No<br>3 I did not use this feature

	42	[exp2]	B) Did you experience any problems when using the TESTS section of the app?	radio (Matrix)<br>1 Yes<br>0 No<br>3 I did not use this feature
	43	[exp3]	C) Did you experience any problems when using the MENSTURAL CYCLE section of the app?	radio (Matrix)<br>1 Yes<br>0 No<br>3 I did not use this feature
	44	[exp4]	D) Did you experience any problems when using the PHYSICAL ACTIVITY section of the app?	radio (Matrix)<br>1 Yes<br>0 No<br>3 I did not use this feature
	45	[exp5]	E) Did you experience any problems when using the TASKS section of the app?	radio (Matrix)<br>1 Yes<br>0 No<br>3 I did not use this feature
	46	[exp6]	F) Did you experience any problems when using the GROUPS section of the app?	radio (Matrix)<br>1 Yes<br>0 No<br>3 I did not use this feature
	47	[exp7]	G) Did you experience any problems when using the LOGGER section of the app?	radio (Matrix)<br>1 Yes<br>0 No<br>3 I did not use this feature
	48	[exp8]	H) Did you experience any problems when using the RESOURCES sections of the app?	radio (Matrix)<br>1 Yes<br>0 No<br>3 I did not use this feature
	49	[exp9]	I) Did you experience any problems when using the COMMODITY ORDERING sections of the app?	radio (Matrix)<br>1 Yes<br>0 No<br>3 I did not use this feature
	50	[exp1_yes]<br>Show the field ONLY if:<br>[exp1] = '1'	Please describe the problems you experienced when using the MENTAL HEALTH section of the app.	text
	51	[exp1_yes1]<br>Show the field ONLY if:<br>[exp1] = '1'	Please rate how much of a problem these issues were for you (select a choice from the dropbox).	dropdown<br>1 Cosmetic problem only: this issue doesn't impact my ability to use this part of the app but it does impact how nice the app looks; only fix it if you have extra time.<br>2 Minor usability problem: this issue has a small impact on my ability to use this part of the app; fixing this should be given low priority.<br>3 Major usability problem: this issue has a large impact on my ability to use this part of the app; important to fix, so should be given high priority.<br>4 Usability catastrophe: this issue keeps me from being able to use this part of the app at all; it is extremely important to fix this before other women use SavvyHER.

	52	[exp2_yes]<br>Show the field ONLY if:<br>[exp2] = '1'	Please describe the problems you experienced when using the TESTS section of the app.	text
	53	[exp2_yes2]<br>Show the field ONLY if:<br>[exp2] = '1'	Please rate how much of a problem these issues were for you (select a choice from the dropbox).	dropdown<br>1 Cosmetic problem only: this issue doesn't impact my ability to use this part of the app but it does impact how nice the app looks; only fix it if you have extra time.<br>2 Minor usability problem: this issue has a small impact on my ability to use this part of the app; fixing this should be given low priority.<br>3 Major usability problem: this issue has a large impact on my ability to use this part of the app; important to fix, so should be given high priority.<br>4 Usability catastrophe: this issue keeps me from being able to use this part of the app at all; it is extremely important to fix this before other women use SavvyHER.
	54	[exp3_yes]<br>Show the field ONLY if:<br>[exp3] = '1'	Please describe the problems you experienced when using the MENSTRUAL CYCLE section of the app.	text
	55	[exp3_yes3]<br>Show the field ONLY if:<br>[exp3] = '1'	Please rate how much of a problem these issues were for you (select a choice from the dropbox).	dropdown<br>1 Cosmetic problem only: this issue doesn't impact my ability to use this part of the app but it does impact how nice the app looks; only fix it if you have extra time.<br>2 Minor usability problem: this issue has a small impact on my ability to use this part of the app; fixing this should be given low priority.<br>3 Major usability problem: this issue has a large impact on my ability to use this part of the app; important to fix, so should be given high priority.<br>4 Usability catastrophe: this issue keeps me from being able to use this part of the app at all; it is extremely important to fix this before other women use SavvyHER.
	56	[exp4_yes]<br>Show the field ONLY if:<br>[exp4] = '1'	Please describe the problems you experienced when using the PHYSICAL ACTIVITY section of the app.	text
	57	[exp4_yes4]<br>Show the field ONLY if:<br>[exp4] = '1'	Please rate how much of a problem these issues were for you (select a choice from the dropbox).	dropdown<br>1 Cosmetic problem only: this issue doesn't impact my ability to use this part of the app but it does impact how nice the app looks; only fix it if you have extra time.<br>2 Minor usability problem: this issue has a small impact on my ability to use this part of the app; fixing this should be given low priority.<br>3 Major usability problem: this issue has a large impact on my ability to use this part of the app; important to fix, so should be given high priority.<br>4 Usability catastrophe: this issue keeps me from being able to use this part of the app at all; it is extremely important to fix this before other women use SavvyHER.
	58	[exp5_yes]<br>Show the field ONLY if:<br>[exp5] = '1'	Please describe the problems you experienced when using the TASKS section of the app.	text

	59	[exp5_yes5]<br>Show the field ONLY if:<br>[exp5] = '1'	Please rate how much of a problem these issues were for you (select a choice from the dropbox).	dropdown<br>1 Cosmetic problem only: this issue doesn't impact my ability to use this part of the app but it does impact how nice the app looks; only fix it if you have extra time.<br>2 Minor usability problem: this issue has a small impact on my ability to use this part of the app; fixing this should be given low priority.<br>3 Major usability problem: this issue has a large impact on my ability to use this part of the app; important to fix, so should be given high priority.<br>4 Usability catastrophe: this issue keeps me from being able to use this part of the app at all; it is extremely important to fix this before other women use SavvyHER.
	60	[exp6_yes]<br>Show the field ONLY if:<br>[exp6] = '1'	Please describe the problems you experienced when using the GROUPS section of the app.	text
	61	[exp6_yes6]<br>Show the field ONLY if:<br>[exp6] = '1'	Please rate how much of a problem these issues were for you (select a choice from the dropbox).	dropdown<br>1 Cosmetic problem only: this issue doesn't impact my ability to use this part of the app but it does impact how nice the app looks; only fix it if you have extra time.<br>2 Minor usability problem: this issue has a small impact on my ability to use this part of the app; fixing this should be given low priority.<br>3 Major usability problem: this issue has a large impact on my ability to use this part of the app; important to fix, so should be given high priority.<br>4 Usability catastrophe: this issue keeps me from being able to use this part of the app at all; it is extremely important to fix this before other women use SavvyHER.
	62	[exp7_yes]<br>Show the field ONLY if:<br>[exp7] = '1'	Please describe the problems you experienced when using the LOGGER section of the app.	text
	63	[exp7_yes7]<br>Show the field ONLY if:<br>[exp7] = '1'	Please rate how much of a problem these issues were for you (select a choice from the dropbox).	dropdown<br>1 Cosmetic problem only: this issue doesn't impact my ability to use this part of the app but it does impact how nice the app looks; only fix it if you have extra time.<br>2 Minor usability problem: this issue has a small impact on my ability to use this part of the app; fixing this should be given low priority.<br>3 Major usability problem: this issue has a large impact on my ability to use this part of the app; important to fix, so should be given high priority.<br>4 Usability catastrophe: this issue keeps me from being able to use this part of the app at all; it is extremely important to fix this before other women use SavvyHER.
	64	[exp8_yes]<br>Show the field ONLY if:<br>[exp8] = '1'	Please describe the problems you experienced when using the RESOURCES section of the app.	text

	65	[exp8_yes8]<br>Show the field ONLY if:<br>[exp8] = '1'	Please rate how much of a problem these issues were for you (select a choice from the dropbox).	dropdown<br>1 Cosmetic problem only: this issue doesn't impact my ability to use this part of the app but it does impact how nice the app looks; only fix it if you have extra time.<br>2 Minor usability problem: this issue has a small impact on my ability to use this part of the app; fixing this should be given low priority.<br>3 Major usability problem: this issue has a large impact on my ability to use this part of the app; important to fix, so should be given high priority.<br>4 Usability catastrophe: this issue keeps me from being able to use this part of the app at all; it is extremely important to fix this before other women use SavvyHER.
	66	[exp9_yes]<br>Show the field ONLY if:<br>[exp9] = '1'	Please describe the problems you experienced when using the COMMODITY ORDERING section of the app.	text
	67	[exp9_yes9]<br>Show the field ONLY if:<br>[exp9] = '1'	Please rate how much of a problem these issues were for you (select a choice from the dropbox).	dropdown<br>1 Cosmetic problem only: this issue doesn't impact my ability to use this part of the app but it does impact how nice the app looks; only fix it if you have extra time.<br>2 Minor usability problem: this issue has a small impact on my ability to use this part of the app; fixing this should be given low priority.<br>3 Major usability problem: this issue has a large impact on my ability to use this part of the app; important to fix, so should be given high priority.<br>4 Usability catastrophe: this issue keeps me from being able to use this part of the app at all; it is extremely important to fix this before other women use SavvyHER.
	68	[app_experience_complete]	Section Header: *Form Status*<br>Complete?	dropdown<br>0 Incomplete<br>1 Unverified<br>2 Complete
Instrument: **Tech Acceptance** (tech_acceptance) Enabled as survey				
	69	[tech_accp1]	Section Header: *TECHNOLOGY ACCEPTANCE Indicate the extent to which you agree with the following statements where 1 = Strongly disagree and 7 = Strongly agree.*<br>Using SavvyHER enables me to monitor my sexual health better than other health apps.	radio (Matrix)<br>1 Strongly disagree<br>2 Somewhat disagree<br>3 Disagree<br>4 Neutral<br>5 Somewhat agree<br>6 Agree<br>7 Strongly agree

	70	[tech_accp2]	Using SavvyHER enables me to monitor my reproductive health better than other health apps.	radio (Matrix)<br>1 Strongly disagree<br>2 Somewhat disagree<br>3 Disagree<br>4 Neutral<br>5 Somewhat agree<br>6 Agree<br>7 Strongly agree
	71	[tech_accp3]	Using SavvyHER improves my sexual and reproductive health experiences.	radio (Matrix)<br>1 Strongly disagree<br>2 Somewhat disagree<br>3 Disagree<br>4 Neutral<br>5 Somewhat agree<br>6 Agree<br>7 Strongly agree
	72	[tech_accp4]	Using SavvyHER improves my mental health.	radio (Matrix)<br>1 Strongly disagree<br>2 Somewhat disagree<br>3 Disagree<br>4 Neutral<br>5 Somewhat agree<br>6 Agree<br>7 Strongly agree
	73	[tech_accp5]	Using SavvyHER improves my physical health.	radio (Matrix)<br>1 Strongly disagree<br>2 Somewhat disagree<br>3 Disagree<br>4 Neutral<br>5 Somewhat agree<br>6 Agree<br>7 Strongly agree
	74	[tech_accp6]	Using SavvyHER enhances my effectiveness in monitoring my sexual and reproductive health.	radio (Matrix)<br>1 Strongly disagree<br>2 Somewhat disagree<br>3 Disagree<br>4 Neutral<br>5 Somewhat agree<br>6 Agree<br>7 Strongly agree
	75	[tech_accp7]	Using SavvyHER enhances my social connectedness with women like me.	radio (Matrix)<br>1 Strongly disagree<br>2 Somewhat disagree<br>3 Disagree<br>4 Neutral<br>5 Somewhat agree<br>6 Agree<br>7 Strongly agree

	76	[tech_accp8]	Using SavvyHER enhances my ability to get support from my network.	radio (Matrix)<br>1 Strongly disagree<br>2 Somewhat disagree<br>3 Disagree<br>4 Neutral<br>5 Somewhat agree<br>6 Agree<br>7 Strongly agree
	77	[tech_accp9]	Using SavvyHER enhances my ability to get support from other women using the SavvyHER app.	radio (Matrix)<br>1 Strongly disagree<br>2 Somewhat disagree<br>3 Disagree<br>4 Neutral<br>5 Somewhat agree<br>6 Agree<br>7 Strongly agree
	78	[tech_accp10]	Using SavvyHER makes my sexual and reproductive health experiences easier.	radio (Matrix)<br>1 Strongly disagree<br>2 Somewhat disagree<br>3 Disagree<br>4 Neutral<br>5 Somewhat agree<br>6 Agree<br>7 Strongly agree
	79	[tech_accp11]	Learning to use SavvyHER was easy for me.	radio (Matrix)<br>1 Strongly disagree<br>2 Somewhat disagree<br>3 Disagree<br>4 Neutral<br>5 Somewhat agree<br>6 Agree<br>7 Strongly agree
	80	[tech_accp12]	I found it easy to get SavvyHER to do what I want it to do	radio (Matrix)<br>1 Strongly disagree<br>2 Somewhat disagree<br>3 Disagree<br>4 Neutral<br>5 Somewhat agree<br>6 Agree<br>7 Strongly agree
	81	[tech_accp13]	My interaction with SavvyHER has been clear and understandable.	radio (Matrix)<br>1 Strongly disagree<br>2 Somewhat disagree<br>3 Disagree<br>4 Neutral<br>5 Somewhat agree<br>6 Agree<br>7 Strongly agree

	82	[tech_accp14]	I found SavvyHER to be flexible to interact with.	radio (Matrix)<br>1 Strongly disagree<br>2 Somewhat disagree<br>3 Disagree<br>4 Neutral<br>5 Somewhat agree<br>6 Agree<br>7 Strongly agree
	83	[tech_accp15]	It was easy for me to become skillful at using SavvyHER.	radio (Matrix)<br>1 Strongly disagree<br>2 Somewhat disagree<br>3 Disagree<br>4 Neutral<br>5 Somewhat agree<br>6 Agree<br>7 Strongly agree
	84	[tech_accp16]	I found SavvyHER easy to use.	radio (Matrix)<br>1 Strongly disagree<br>2 Somewhat disagree<br>3 Disagree<br>4 Neutral<br>5 Somewhat agree<br>6 Agree<br>7 Strongly agree
	85	[feedbk]	Is there anything the SavvyHER team should know to make the app more helpful for Black women?	text
	86	[tech_acceptance_complete]	Section Header: *Form Status*<br>Complete?	dropdown<br>0 Incomplete<br>1 Unverified<br>2 Complete
